# Defective mitochondrial fission augments NLRP3 inflammasome activation

**DOI:** 10.1038/srep15489

**Published:** 2015-10-22

**Authors:** Sangjun Park, Ji-Hee Won, Inhwa Hwang, Sujeong Hong, Heung Kyu Lee, Je-Wook Yu

**Affiliations:** 1 Department of Microbiology, Institute for Immunology and Immunological Diseases, Brain Korea 21 PLUS Project for Medical Science, Yonsei University College of Medicine, Seoul 120-752, Korea; 2 Laboratory of Host Defenses, Graduate School of Medical Science and Engineering, Korea Advanced Institute of Science and Technology, Daejeon 305-701, Korea

## Abstract

Despite the fact that deregulated NLRP3 inflammasome activation contributes to the pathogenesis of chronic inflammatory or metabolic disorders, the underlying mechanism by which NLRP3 inflammasome signaling is initiated or potentiated remains poorly understood. Much attention is being paid to mitochondria as a regulator of NLRP3 inflammasome activation, but little is known about the role of mitochondrial dynamics for the inflammasome pathway. Here, we present evidence that aberrant mitochondrial elongation caused by the knockdown of dynamin-related protein 1 (Drp1) lead to a marked increase in NLRP3-dependent caspase-1 activation and interleukin-1-beta secretion in mouse bone marrow-derived macrophages. Conversely, carbonyl cyanide *m*-chlorophenyl hydrazone, a chemical inducer of mitochondrial fission, clearly attenuated NLRP3 inflammasome assembly and activation. Augmented activation of NLRP3 inflammasome by mitochondrial elongation is not resulted from the increased mitochondrial damages of Drp1-knockdown cells. Notably, enhanced extracellular signal-regulated kinase (ERK) signaling in Drp1-knockdown macrophages is implicated in the potentiation of NLRP3 inflammasome activation, possibly via mediating mitochondrial localization of NLRP3 to facilitate the assembly of NLRP3 inflammasome. Taken together, our results provide a molecular insight into the importance of mitochondrial dynamics in potentiating NLRP3 inflammasome activation, leading to aberrant inflammation.

Inflammasomes are caspase-1-activating multi-protein complex, comprised of sensor protein such as NOD-like receptor family, pyrin domain-containing 3 (NLRP3) or NLR family, CARD-containing 4 (NLRC4), adaptor protein apoptosis-associated speck-like protein containing a caspase-recruitment domain (ASC) and effector protein procaspase-1^1^. The assembly of inflammasome components upon sensing of microbial infection or tissue injury mediates caspase-1-dependent processing and secretion of interleukin (IL)-1β or IL-18 to provide host innate immune protection^1^,^2^. Despite the fact that deregulated NLRP3 inflammasome activation potentially contributes to the pathogenesis of chronic inflammatory or metabolic disorders such as type 2 diabetes and Alzheimer’s disease^3^,^4^, the underlying mechanism by which NLRP3 inflammasome signaling is initiated or potentiated remains still elusive.

The role of mitochondria in regulating the activation of the NLRP3 inflammasome has garnered much interest^5^,^6^. The accumulation of damaged mitochondria might trigger the activation of the NLRP3 inflammasome by releasing mitochondrial reactive oxygen species (mROS) or mitochondrial DNA (mtDNA) into the cytosol^5^,^7^. Moreover, mitochondria could serve as a molecular platform for the NLRP3 inflammasome in specific cellular contexts; for instance, mitochondrial outer membrane proteins such as mitochondrial anti-viral signaling protein (MAVS) or mitofusin 2 (Mfn2) could recruit NLRP3 to assemble the inflammasome complex in response to viral infection^8^,^9^,^10^.

Mitochondria are dynamic organelles that continuously fuse and divide to control their size, morphology, mtDNA integrity, and cellular redox state^11^. Mitochondrial dynamics are well-balanced with mitochondrial biogenesis and degradation (mitophagy) to maintain mitochondrial homeostasis^12^. The regulation of mitochondrial dynamics is controlled by specific proteins for mitochondrial fission or fusion, such as dynamin-related protein 1 (Drp1) and fission protein 1 (Fis1) for fission, and Mfn1, Mfn2, and optic atrophy gene 1 (Opa1) for fusion^12^. An imbalance in mitochondrial dynamics, caused by loss-of-function mutations in proteins that make up the mitochondrial fission and fusion machinery, not only results in the impairment of mitochondrial or cellular functions such as energy production, mtDNA integrity, and cell proliferation, but also causes many complex disorders including Parkinson’s or Alzheimer’s disease^11^,^12^.

The role of mitochondrial dynamics in the regulation of innate immune responses was recently discovered: through its association with MAVS, Mfn2 interferes with MAVS-mediated anti-viral signaling in response to viral infection^13^. Other studies, on the contrary, revealed that mitochondrial elongation is required for MAVS signaling in an Mfn1-dependent manner^14^,^15^. These findings indicate that mitochondrial fusion facilitates type I interferon (IFN) production in response to virus infection. However, the putative role of mitochondrial dynamics in inflammasome signaling remains poorly described. Intriguingly, elongated mitochondria, formed by a failure of Drp1 to localize into mitochondria or by decreased Drp1 expression in the Alzheimer’s disease patients, accumulated in the perinuclear region^16^,^17^, where the indications of NLRP3 inflammasome assembly including ASC speck-like aggregates and association between NLRP3 and ASC have also been observed^18^,^19^,^20^. We therefore attempted to investigate whether altered mitochondrial dynamics due to knockdown of Drp1 could modulate the assembly and activation of the NLRP3 inflammasome.

## Results

### Knockdown of Drp1 leads to mitochondrial elongation and increases susceptibility to caspase-1-dependent cell death

**Figure 1:**
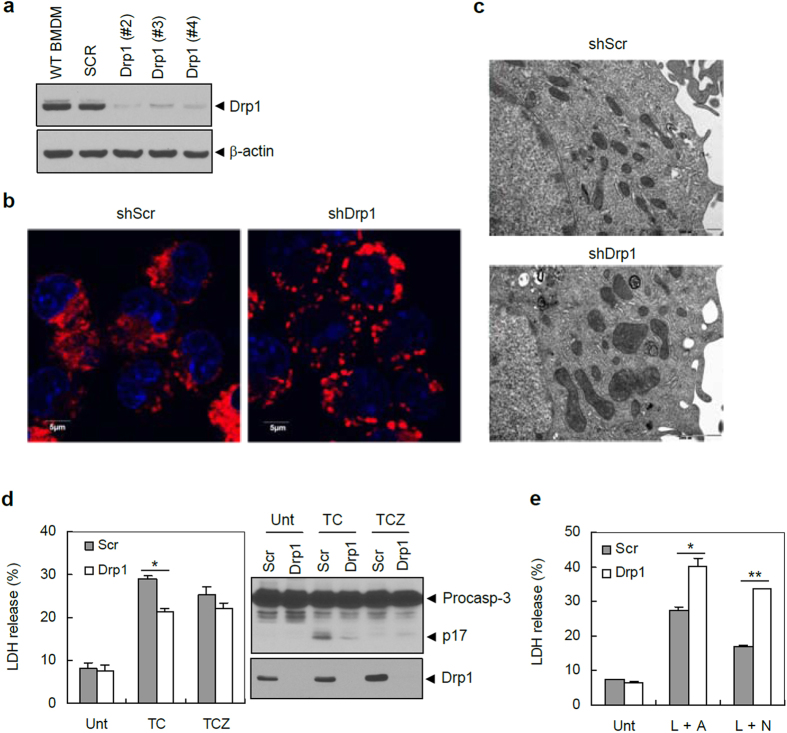
Knockdown of Drp1 leads to mitochondrial elongation and increases pyroptotic cell death of macrophages. (**a**) Expression levels of Drp1 in wild-type mouse BMDMs and scrambled shRNA- (Scr) or Drp1-specific shRNA-expressing (Drp1) BMDMs. (**b**) Confocal images of shScr or shDrp1 BMDMs stained with MitoTracker Red. The blue signal represents nuclear fluorescence. Scale bars, 5 μm. (**c**) Transmission electron microscopy (TEM) images of shScr or shDrp1 BMDMs. Scale bars, 2 μm. Magnification, 20,000×. (**d**,**e**) ShScr or shDrp1 BMDMs were stimulated with TNF-α and CHX in the absence (TC) or presence (TCZ) of zVAD-fmk, or primed with LPS, followed by ATP (L + A) or nigericin (L + N) treatment. Cells were then assayed for LDH release and cellular lysates were immunoblotted with the indicated antibodies. Asterisks indicate significant differences (*n *= 6, *p<0.0005, d; *n *= 6, *p < 0.0005, **p < 0.0001, e).

To generate macrophages with stable knockdown of Drp1, lentiviral particles expressing non-targeting (scrambled) or Drp1-targeting short hairpin RNA (shRNA) were used to infect mouse bone marrow-derived macrophages (BMDMs). Drp1 expression was largely abolished in the selected clones of shDrp1 BMDMs (Fig. 1a). As expected, mitochondria from Drp1-knockdown macrophages displayed an elongated or enlarged shape as observed by confocal and transmission electron microscopy (Fig. 1b,c). Because mitochondria play an important role in regulating programmed cell death, we first determined the sensitivity of both macrophages to apoptotic, necroptotic, or pyroptotic cell death stimulation. In agreement with previous reports, shDrp1 cells containing elongated mitochondria exhibited significantly decreased apoptosis and caspase-3 processing compared to shScr BMDMs upon TNF-α-cycloheximide (TC) stimulation (Fig. 1d)^21^,^22^. On the contrary, both types of macrophages exhibited similar RIP-3-dependent necroptosis in response to TNF-α-cycloheximide-zVAD (TCZ) stimulation (Fig. 1d). The requirement of Drp1 for necroptosis is still controversial^23^,^24^; the precise role of Drp1 in necroptosis remains to be clarified.

Of more interest, NLRP3 inflammasome-mediated pyroptotic cell death was significantly higher in shDrp1 BMDMs than in control BMDMs upon NLRP3-activating LPS/ATP or LPS/nigericin stimulation (Fig. 1e). Supporting this data, Drp1-knockdown BMDMs showed a potentiated cell death upon ATP or nigericin stimulation, but a decreased cell death upon TC stimulation, as determined by propidium iodide (PI) staining (Supplementary Fig. S1a). In addition, TC-induced apoptosis was decreased in Drp1-knockdown BMDMs (Supplementary Fig. S1b). Similar to the previous reports^25^,^26^, LPS/nigericin stimulation caused a NLRP3-dependent cell death (Supplementary Fig. S1c), and ATP- or nigericin-triggered cell death was significantly attenuated by YVAD, a specific caspase-1 inhibitor (Supplementary Fig. S1d and S1e). These findings raise the possibility that caspase-1/inflammasome signaling might be more potent in shDrp1 BMDMs containing elongated mitochondria than in shScr BMDMs.

### Mitochondrial elongation, but not fission, potentiates NLRP3 inflammasome activation

**Figure 2:**
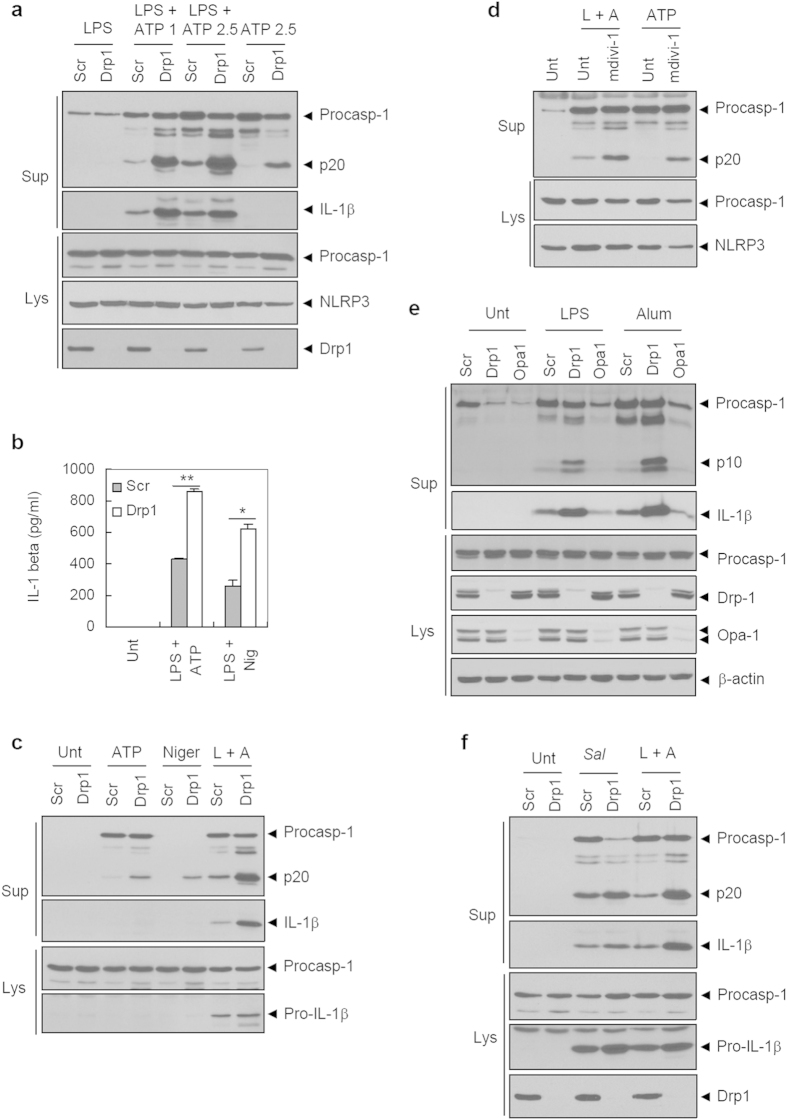
Mitochondrial elongation potentiates NLRP3 inflammasome activation. (**a**) ShScr or shDrp1 BMDMs were primed with LPS, followed by ATP (1 or 2.5 mM) treatment as indicated. (**b**) ShScr or shDrp1 BMDMs were primed with LPS, followed by ATP (2.5 mM) or nigericin (5 μM) treatment. Culture supernatants were assayed for extracellular IL-1β by ELISA. Asterisks indicate significant differences (*n *= 3, *p < 0.05, **p < 0.005). (**c**) ShScr or shDrp1 BMDMs were untreated or treated with ATP (2.5 mM, 40 min) in the presence or absence of LPS priming, or treated with nigericin (5 μM, 40 min). (**d**) Wild-type BMDMs were untreated or treated with LPS in the presence or absence of mdivi-1 (25 μM) pretreatment, followed with ATP treatment. (**e**) PMA-differentiated shScr, shDrp1 or shOpa1 THP-1 cells were stimulated with LPS or Alum. (**f**) ShScr or shDrp1 BMDMs were untreated, infected with *S. typhimurium* (*Sal*, MOI 20) as described in Methods or treated with LPS, followed by ATP as in (**c**). (**a**,**c**–**f**) Culture supernatants (Sup) and cellular lysates (Lys) were immunoblotted with the indicated antibodies.

We next examined whether mitochondrial elongation indeed affects the NLRP3-dependent activation of caspase-1. In LPS-primed BMDMs, the knockdown of Drp1 induced much stronger caspase-1 processing in response to ATP stimulation (Fig. 2a and Supplementary Fig. S2). Supporting this finding, IL-1β secretion was also remarkably increased in Drp1-knockdown macrophages upon LPS/ATP or LPS/nigericin stimulation (Fig. 2b). Notably, only ATP or nigericin stimulation (signal 2) without LPS priming (signal 1) is sufficient to activate caspase-1 activation in Drp1-knockdown BMDMs, but not in shScr BMDMs (Fig. 2a,c). Furthermore, specific inhibition of Drp1 by mitochondrial division inhibitor-1 (mdivi-1) also consistently potentiated LPS/ATP- or ATP-mediated caspase-1 activation in wild-type BMDMs (Fig. 2d). To validate this finding, we checked the role of altered mitochondrial dynamics for the inflammasome activity in human monocytic THP-1 cells stably expressing non-targeting, Drp1-targeting or Opa1-targeting shRNA. Supporting above-mentioned results in mouse BMDMs, both caspase-1 activation and IL-1β secretion was markedly increased in Drp1-knockdown THP-1 cells, but not in Opa1-knockdown cells, compared to control scrambled cells in response to LPS or Alum stimulation (Fig. 2e). On the other hand, the NLRC4-dependent caspase-1 activation by *Salmonella* infection was not considerably affected by the knockdown of Drp1 in macrophages (Fig. 2f).

**Figure 3:**
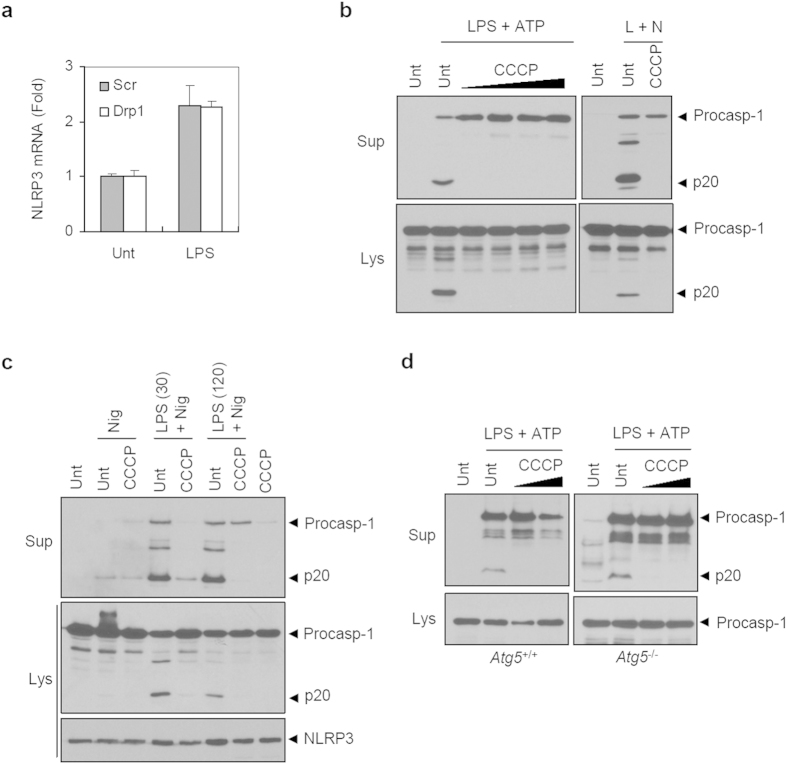
CCCP attenuates NLRP3 inflammasome activation. (**a**) ShScr or shDrp1 BMDMs were treated with LPS (0.5 μg/ml, 3 h), and mRNA production was determined by quantitative real-time PCR as described in Methods (*n *= 3). (**b**) Wild-type BMDMs were treated with LPS in the presence of CCCP (1–40 μM, left panel; 20 μM, right panel), followed by ATP (left) or nigericin (right) treatment. (**c**) Immortalized NLRP3-reconstituted macrophages (N1–8) were unprimed or primed with LPS (30 min or 120 min) in the presence of CCCP (20 μM), followed by nigericin treatment as indicated. (**d**) Wild-type or *Atg5*-deficient BMDMs were treated with LPS in the presence of CCCP (5 or 10 μM) pretreatment as indicated, followed with ATP treatment. (**b**–**d**) Culture supernatants (Sup) and cellular lysates (Lys) were immunoblotted with the indicated antibodies.

To examine whether the potentiated NLRP3 inflammasome activation in Drp1-knockdown macrophages is due to an increase in the transcriptional induction of NLRP3, we determined the mRNA levels of *Nlrp3* upon LPS stimulation. Consequently, Drp1-knockdown cells exhibited no significant difference in the mRNA production of *Nlrp3* compared to control scrambled macrophages in response to LPS stimulation (Fig. 3a).

The protonophore carbonyl cyanide *m*-chlorophenyl hydrazone (CCCP) has been shown to induce mitochondrial fission or fragmentation in a Drp1-dependent manner^27^. Supporting our data, CCCP treatment largely impaired caspase-1 activation in LPS/ATP or LPS/nigericin-stimulated BMDMs (Fig. 3b). To examine whether CCCP-induced attenuation of caspase-1 activation stems from the reduction in the expression of NLRP3, we performed a similar experiment in NLRP3-reconstituted macrophages (N1–8) in which NLRP3 expression is not regulated by TLR-NF-κB signaling^28^. Consistently, CCCP abolished LPS/nigericin-triggered caspase-1 activation in NLRP3-stably expressing macrophages (Fig. 3c), demonstrating that CCCP-mediated inhibition of NLRP3 inflammasome is not resulted from the reduced NLRP3 expression. Contrary to our data, previous studies demonstrated that CCCP promotes IL-1β secretion possibly via the production of mROS^5^,^29^. However, CCCP did not induce mROS production in our experiment (Supplementary Fig. S3).

Mitochondrial uncoupler CCCP was also reported to induce parkin-mediated mitophagy^30^. We thus examined the inhibitory effect of CCCP on the NLRP3 inflammasome in *Atg5*-deficient BMDMs to determine the possibility that mitophagy could mediate the CCCP-induced inhibition. However, CCCP-induced inhibition of caspase-1 activation by LPS/ATP was also evident in *Atg5*^−/−^ BMDMs (Fig. 3d). These data suggest that CCCP-induced mitochondrial fission or fragmentation does not promote but suppress NLRP3 inflammasome activation in a mitophagy-independent manner.

### Mitochondrial elongation potentiates NLRP3 inflammasome assembly

**Figure 4:**
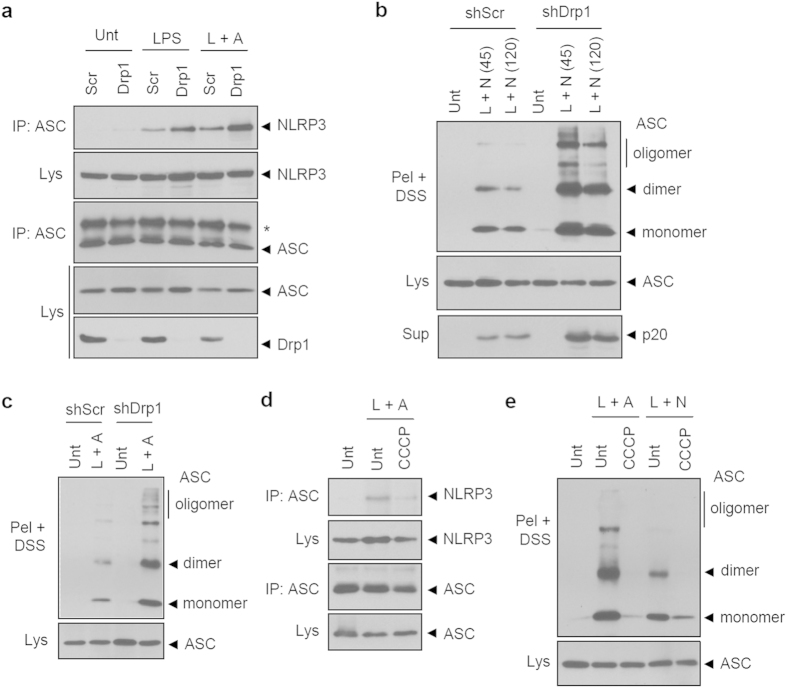
Mitochondrial elongation facilitates the assembly of the NLRP3 inflammasome. (**a**) ShScr or shDrp1 BMDMs were untreated or treated with LPS, followed with ATP (2.5 mM, 30 min) as indicated. Soluble lysates were immunoprecipitated with anti-ASC antibody, and the immunoprecipitates or lysates were immunoblotted with the indicated antibodies. (**b**,**c**) ShScr or shDrp1 BMDMs were untreated or treated with LPS, followed by treatment with nigericin (45 min or 120 min, (**b)**) or ATP (2 mM, 45 min, (**c)**). ASC oligomeric structures were assayed by DSS-mediated cross-linking. (**d**,**e**) Wild-type BMDMs were untreated or treated with LPS in the presence or absence of CCCP (5 μM) for 3 h, followed by ATP or nigericin as indicated. NLRP3-ASC association (**d**) and ASC oligomerization (**e**) were assayed as described in (**a**) and (**b**).

We, next, examined the role of mitochondrial dynamics in the assembly of the NLRP3 inflammasome as determined by NLRP3-ASC interaction and ASC oligomerization. Both events are essential and prerequisite steps for the activation of NLRP3 inflammasome^18^,^31^. LPS priming substantially promoted the association of NLRP3 with ASC, but the NLRP3-ASC interaction was much stronger in Drp1-knockdown BMDMs (Fig. 4a). In a similar manner, ASC oligomerization, triggered by LPS/nigericin or LPS/ATP stimulation, was much more potent in shDrp1 BMDMs than in shScr BMDMs (Fig. 4b,c). On the contrary, CCCP treatment to induce mitochondrial fission considerably attenuated the NLRP3-ASC interaction and ASC oligomerization (Fig. 4d,e). These findings indicate that mitochondrial elongation by Drp1 knockdown facilitates the NLRP3-ASC association and the subsequent ASC oligomerization, although the underlying mechanism by which mitochondrial elongation controls the assembly of the NLRP3 inflammasome remains uncertain.

### Augmented NLRP3 inflammasome activation in Drp1-knockdown macrophages is independent of the increased mitochondrial damages

**Figure 5:**
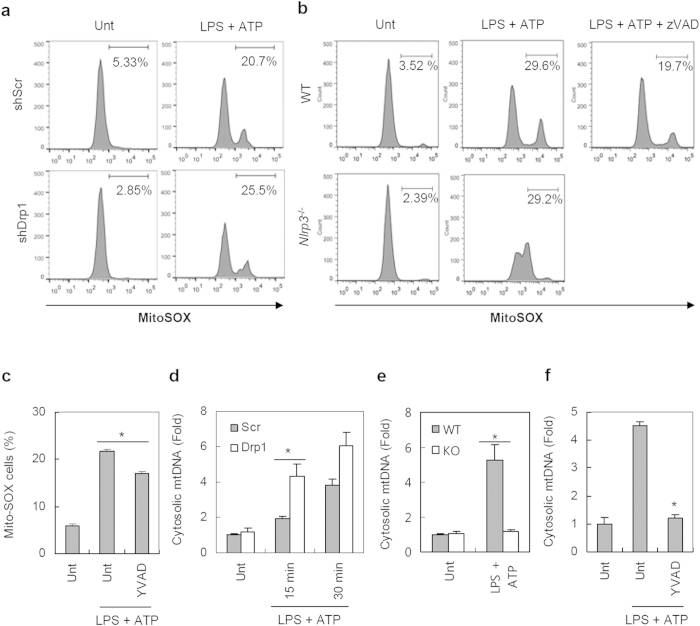
Drp1-knockdown BMDMs exhibit increased mROS production and mtDNA release. (**a**) Scrambled or Drp1-knockdown BMDMs were untreated or treated with LPS, followed by treatment with ATP (2.5 mM, 30 min). (**b**) Wild-type or *Nlrp3*^−/−^ BMDMs were untreated or treated with LPS (0.25 μg/ml, 2 h) in the presence or absence of zVAD-fmk (20 μM, 30 min pretreatment before LPS) as indicated, followed with ATP. (**a**,**b**) Cells were stained with MitoSOX and analyzed by flow cytometry. (**c**) ShDrp1 BMDMs were primed with LPS in the presence or absence of YVAD-cmk, followed by treatment with ATP. Cells were stained with MitoSOX and analyzed by flow cytometry. MitoSOX-positive cells were plotted. Asterisk indicates a significant difference (*n *= 4, *p < 0.01). (**d**,**e**) Scrambled or Drp1-knockdown BMDMs (**d**) and wild-type or *Nlrp3*^−/−^ BMDMs (**e**) were primed with LPS, followed by treatment with ATP (15 or 30 min, (**d)** 30 min, (**e)**) as indicated. Cytosolic mtDNA was quantified as described in Methods. Asterisks indicate significant differences (*n *= 3, *p < 0.05). (f) ShDrp1 BMDMs were primed with LPS in the presence or absence of YVAD-cmk, followed by treatment with ATP. Cytosolic mtDNA was then assayed. Asterisk indicates a significant difference in comparison to LPS/ATP-treated samples (*n *= 3, *p < 0.0001).

To provide molecular insights into how mitochondrial elongation potentiates the activation of the NLRP3 inflammasome, we determined the generation of mitochondria-derived products such as mROS and mtDNA in both types of BMDMs. LPS/ATP stimulation caused a substantial increase in mROS-producing mitochondria, as indicated by mROS-sensitive MitoSOX staining (Fig. 5a). Upon LPS/ATP stimulation, Drp1-knockdown BMDMs exhibited slightly elevated mROS production compared to shScr BMDMs (Fig. 5a). However, LPS/ATP-mediated mROS production was partially impaired by the absence of NLRP3 or by the presence of the pan-caspase inhibitor zVAD (Fig. 5b). Furthermore, a selective caspase-1 inhibitor YVAD significantly reduced mROS production by LPS/ATP stimulation (Fig. 5c and Supplementary Fig. S4). In this regard, we cannot exclude the possibility that the increased mROS production reflects, at least in part, the augmented caspase-1 activation in Drp1-knockdown macrophages.

ATP stimulation of LPS-primed BMDMs caused a robust release of mtDNA into the cytosol (Fig. 5d). Of note, mtDNA release was slightly but significantly higher in shDrp1 BMDMs than in shScr BMDMs, especially at 15 min of ATP stimulation. However, LPS/ATP-induced cytoplasmic release of mtDNA was completely abolished in *Nlrp3*^−/−^ BMDMs (Fig. 5e), indicating that mtDNA release might depend on caspase-1 activation or the presence of NLRP3, as previously suggested^7^. This finding led us to infer that the increased mtDNA release is not responsible for the stronger caspase-1 activation in Drp1-knockdown BMDMs. Indeed, the inhibition of caspase-1 by YVAD largely abolished LPS/ATP-promoted mtDNA release (Fig. 5f).

**Figure 6:**
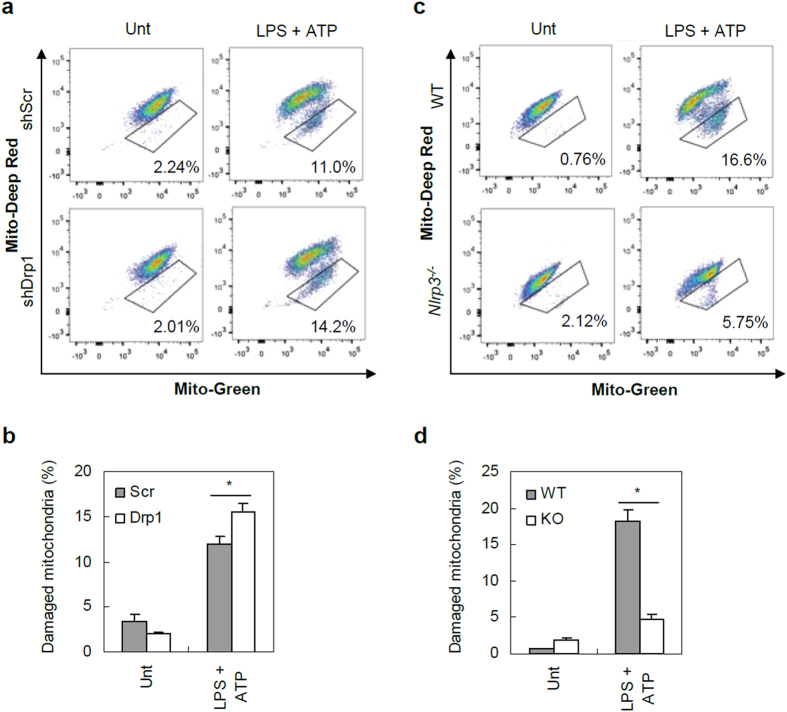
Mitochondrial damage reflects inflammasome activation. ShScr or shDrp1 BMDMs (**a**,**b**) and wild-type or *Nlrp3*^−/−^ BMDMs (**c**,**d**) were untreated or treated with LPS, followed with ATP (2.5 mM, 30 min). Cells were costained with MitoTracker Green and MitoTracker Deep Red, and then analyzed by flow cytometer. Representative histograms of four- or three-independent experiments were presented as in (**a**) and (**c**). (**b**,**d**) Relative cell populations inside the box from independent experiments were plotted. Asterisk indicates a significant difference (*n *= 4, *p < 0.01, b; *n *= 3, *p < 0.05, d).

To provide more evidence whether the increased mitochondrial damage contributes into the potentiated NLRP3 inflammasome activation in Drp1-knockdown cells, we determined another indication of mitochondrial damage by costaining the cells with MitoTracker Green and MitoTracker Deep Red to measure the dissipation of mitochondrial membrane potential. As expected, LPS/ATP stimulation caused a substantial increase in the damaged mitochondria-containing cell population as indicated by the reduction of MitoTracker Deep Red signal (Fig. 6a). Indeed, LPS/ATP-mediated mitochondrial damage was significantly higher in shDrp1 BMDMs than in shScr cells (Fig. 6b). However, in agreement with a recent report^32^, LPS/ATP-induced mitochondrial damage was largely impaired in *Nlrp3*^−/−^ BMDMs (Fig. 6c,d). This finding also supports the idea that increased mitochondrial damage, as well as the presence of mtDNA, might not be a main contributor to potentiating NLRP3 inflammasome activation, but rather a consequence of augmented caspase-1 activation in shDrp1 cells. Therefore, we reasoned that some mechanistic basis other than that of increased mitochondrial damage is required to potentiate NLRP3 inflammasome activation in Drp1-knockdown macrophages.

### Knockdown of Drp1 induces constitutive activation of ERK to facilitate the assembly of the NLRP3 inflammasome

**Figure 7:**
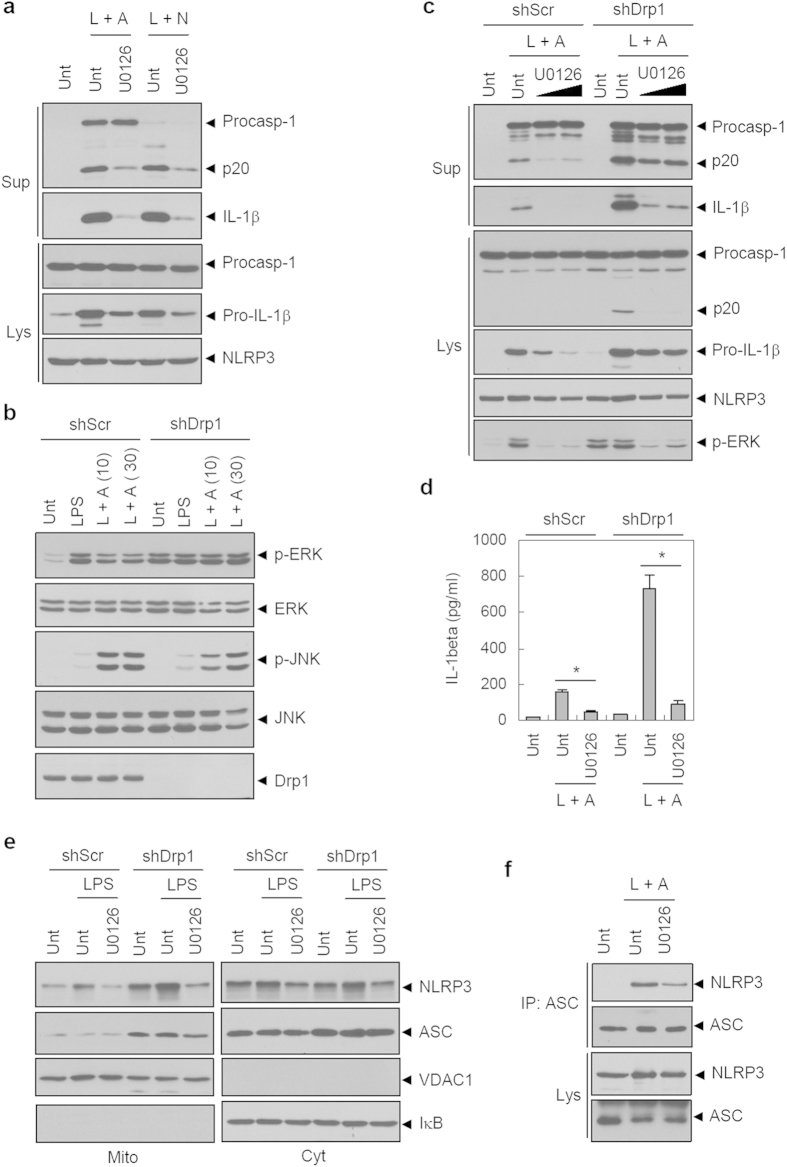
Mitochondrial elongation potentiates NLRP3 inflammasome in an ERK-dependent manner. (**a**) Wild-type BMDMs were untreated or treated with LPS in the presence or absence of U0126 (10 μM, 30 min pretreatment before LPS), followed with ATP or nigericin treatment as indicated. (**b**) ShScr or shDrp1 BMDMs were untreated or treated with LPS, followed by ATP (2.5 mM, 10 min or 30 min) treatments as indicated. (**c**,**d**) ShScr or shDrp1 BMDMs were untreated or treated with LPS in the presence or absence of U0126 (10 μM, 30 min or 120 min (**c**) or 30 min (**d**) pretreatment), followed by treatment with ATP. (**d**) Culture supernatants were assayed for extracellular IL-1β by ELISA. Asterisks indicate significant difference from LPS/ATP-only treated samples in shScr or shDrp1 cells (*n *= 3, *p < 0.005). (**e**) ShScr or shDrp1 BMDMs were untreated or treated with LPS in the presence or absence of U0126 pretreatment (10 μM, 2 h). Cellular lysates were fractionated into mitochondria-enriched (Mito) or cytosolic (Cyt) fractions, and each of the fractionated samples was immunoblotted. (**f**) Wild-type BMDMs were untreated or treated with LPS in the presence of U0126 pretreatment (10 μM, 30 min), followed by treatment with ATP. Soluble lysates were immunoprecipitated with the anti-ASC antibody, and the immunoprecipitates or lysates were immunoblotted.

Recently, it was proposed that extracellular signal-regulated kinase (ERK) signaling is implicated in the LPS-induced priming of NLRP3, required for inflammasome activation^33^. Indeed, inhibition of the ERK pathway by U0126, a selective inhibitor of MEK1 and MEK2, markedly diminished ATP- or nigericin-mediated caspase-1 activation in wild-type BMDMs (Fig. 7a). Interestingly, mitochondrial localization of ERK and its upstream MAPK/ERK kinase (MEK) was previously reported^34^,^35^. We thus test whether ERK signaling might be implicated in augmented NLRP3 inflammasome activation in Drp1-knockdown macrophages.

LPS stimulation caused robust ERK1 and ERK2 phosphorylation in shScr BMDMs (Fig. 7b). Intriguingly, Drp1-knockdown BMDMs displayed a constitutive basal phosphorylation of ERK, but not of c-Jun N-terminal kinase (JNK), even without LPS stimulation (Fig. 7b). Supporting this observation, CCCP attenuated LPS- or LPS/ATP-mediated ERK phosphorylation (Supplementary Fig. S5). ERK phosphorylation in shDrp1 BMDMs was also attenuated by U0126 (Fig. 7c), and U0126 treatment significantly inhibited LPS/ATP-mediated caspase-1 activation and IL-1β secretion in shDrp1 BMDMs (Fig. 7c,d). These observations demonstrate that ERK signaling is implicated in the augmented NLRP3 inflammasome activation seen in Drp1-knockdown macrophages.

Because mitochondria could serve as a platform to assemble inflammasome components^5^,^8^,^9^,^10^, we assessed the subcellular distribution of NLRP3 inflammasome components. In shScr BMDMs, LPS priming increased mitochondrial distribution of NLRP3, which is inhibited by U0126 (Fig. 7e). Furthermore, the association of NLRP3 with ASC was also partially attenuated by U0126 treatment (Fig. 7f), indicating that ERK-dependent mitochondrial localization of NLRP3 is important for the assembly of the NLRP3 inflammasome. Consistent with this, in addition to ERK phosphorylation, the basal mitochondrial localization of NLRP3 was increased in unstimulated shDrp1 cells compared to shScr cells (Fig. 7e). Unexpectedly, ASC levels in mitochondria were also higher in shDrp1 cells to provide a more favorable cellular context for the assembly of the NLRP3 inflammasome. Collectively, our data present evidence that mitochondrial elongation induces the activation of the ERK pathway, which is important for the recruitment of NLRP3 into mitochondria, and this, in turn, facilitates the assembly and activation of the NLRP3 inflammasome.

## Discussion

Mitochondrial dynamics play an important role in maintaining the size, number, and integrity of mitochondria during cell division^36^. Genetic ablations or mutations in the mitochondrial dynamics machinery have shown severe defects in brain development or embryonic lethality^12^,^27^. Of particular interest, a loss-of-function mutation in Drp1 or a failure of Drp1 to locate into mitochondria causes mitochondrial elongation, resulting in the defective brain development, optic atrophy and neurodegeneration^16^,^37^,^38^. Supporting this, fibroblasts from Alzheimer’s disease patients showed a significant decrease in Drp1 expression and consequently hyperfused mitochondria^17^. Considering that inflammasome-mediated neuroinflammation significantly contributes to the pathogenesis of neurodegenerative diseases like Alzheimer’s disease^39^, it is of great significance to decipher the role of Drp1-mediated defective mitochondrial dynamics in inflammasome signaling.

**Figure 8:**
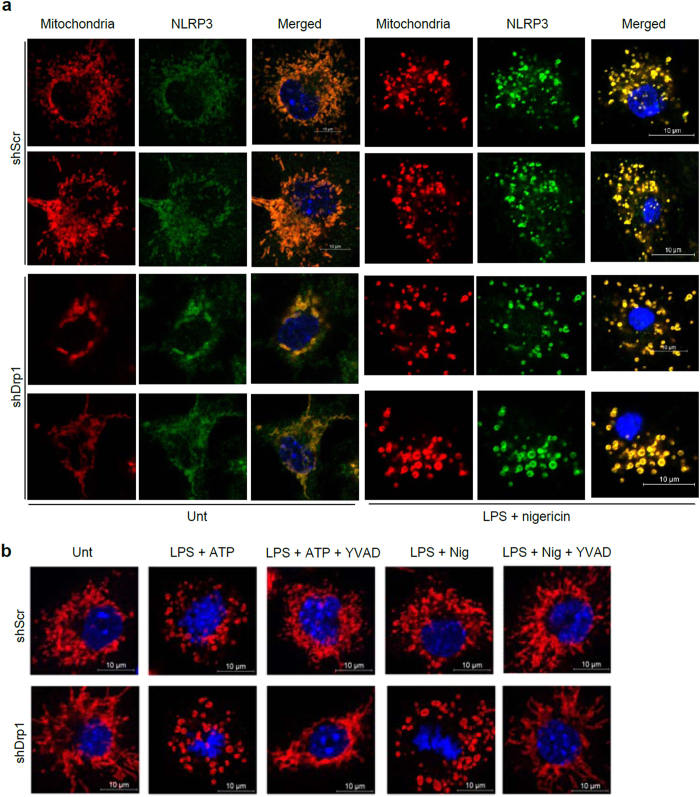
Mitochondrial fragmentation induced by NLRP3 inflammasome activation. (**a**) Confocal images of shScr or shDrp1 BMDMs left untreated or treated with LPS (0.25 μg/ml, 3 h), followed with nigericin (5 μM, 1 h) stimulation. Immunofluorescence assays were performed using anti-Tom20 antibody (red) and anti-NLRP3 antibody (green). (**b**) ShScr or ShDrp1 BMDMs were primed with LPS in the presence of YVAD (20 μM), followed by ATP (2 mM, 45 min) or nigericin (5 μM, 45 min) treatment. Cells were stained with anti-Tom20 antibody (red) and analyzed by confocal microscopy as in (**a**). (**a**,**b**) Blue signal represents nuclear fluorescence. Scale bars, 10 μm.

In addition to generating cellular energy, mitochondria participate in several intracellular signaling pathways such as apoptosis and anti-viral responses^40^. Furthermore, recent studies proposed that indicators of mitochondrial damage such as mROS production, cytosolic release of mtDNA, and the dissipation of mitochondrial membrane potential are crucial common factors for triggering NLRP3 inflammasome activation^5^,^7^. Our data also show that Drp1-knockdown macrophages generated increased levels of mitochondrial damage-associated indicators under NLRP3-stimulating condition. However, these indications were, at least in part, caspase-1 activation-dependent, and could be potentiated by caspase-1/inflammasome activation (Figs 5 and 6). We therefore inferred that increased mitochondrial damage does not fully explain the augmented caspase-1 activation in Drp1-knockdown macrophages. In support of this idea, sustained LPS/nigericin stimulation caused robust mitochondrial fragmentation in scrambled and Drp1-knockdown BMDMs (Fig. 8a), indicating that mitochondrial fragmentation might be the consequence of caspase-1 activation. Furthermore, YVAD, a selective caspase-1 inhibitor, clearly prevented mitochondrial fragmentation induced by LPS/ATP or LPS/nigericin stimulation (Fig. 8b and Supplementary Fig. S6). In addition, CCCP treatment to induce mitochondrial fission or fragmentation efficiently abolished NLRP3-dependent caspase-1 activation and NLRP3 inflammasome assembly in our data (Figs 3 and 4). Although CCCP was previously shown to promote or inhibit NLRP3 inflammasome^5^,^10^, our data emphasizes that CCCP-mediated mitochondrial fission or fragmentation appears to attenuate the assembly and the activation of NLRP3 inflammasome in a transcription- or mitophagy-independent manner.

The role of mitochondrial dynamics in inflammasome signaling has not been much explored until recently. Ichinohe *et al.* first demonstrated that Mfn2 is required for the full activation of NLRP3 inflammasome through the formation of the NLRP3-Mfn2-MAVS complex upon RNA virus infection^10^, suggesting that mitochondrial fusion is favorable to NLRP3 inflammasome assembly. In contrast, Wang *et al.* recently showed that RNA viral infection promotes the assembly of the RIP1-RIP3 complex, which phosphorylates Drp1, leading to mitochondrial fission and the subsequent activation of the NLRP3 inflammasome^29^. This study proposed that Drp1-mediated mitochondrial fission increases mitochondrial damage, resulting in the activation of the NLRP3 inflammasome upon viral infection. These recent findings are contradictory in terms of the role of mitochondrial dynamics on the viral RNA-mediated NLRP3 inflammasome, but our study results show that Drp1-knockdown cells containing elongated mitochondria induce augmented NLRP3 activation in response to classical ATP or nigericin stimulation.

Interestingly, a previous report also demonstrated that Drp1 deficiency had no effect on the IL-1β secretion of BMDMs in response to LPS/ATP or LPS/nigericin stimulation^29^. They suggested that ATP or nigericin promoted mROS production or mitochondrial damages in a Drp1-independent manner. In this regard, ATP- or nigericin-induced NLRP3 inflammasome activation is independent of Drp1 presence. However, our results showed that augmented NLRP3 inflammasome activation by Drp1 knockdown might be explained by the increased association of inflammasome components, rather than by the production of mROS or mitochondrial damages. Furthermore, our data strongly suggest that mitochondrial elongation provides a favorable cellular context for NLRP3 activation, while mitochondrial fission or fragmentation is rather a consequence of inflammasome activation.

Our data also present that the ERK pathway is a critical priming event for the activation of NLRP3 inflammasome. We revealed that enhanced ERK signaling mediates the recruitment of NLRP3 into mitochondria, facilitating the assembly of the NLRP3 inflammasome in Drp1-knockdown macrophages. Although the question of how mitochondrial elongation induces ERK activation requires more clarification, the ERK pathway could be an effective target to alleviate excessive NLRP3 inflammasome activation resulting from aberrant mitochondrial elongation. In conclusion, our data demonstrate that mitochondrial elongation caused by the decreased expression of the fission protein Drp1 creates a favorable cellular context for NLRP3 inflammasome activation in an ERK-dependent manner, thereby leading to diseases associated with deregulated inflammation.

## Methods

### Reagents and antibodies

LPS, ATP, nigericin, cycloheximide (CHX), CCCP, mdivi-1, propidium iodide (PI), U0126, and Drp1-targeting or non-targeting shRNA lentiviral plasmids were purchased from Sigma. Alum was purchased from InvivoGen. Ac-YVAD-chloromethylketone (Ac-YVAD-cmk) and z-VAD-fluoromethylketone (zVAD-fmk) were obtained from Bachem. Mouse IL-1β enzyme-linked immunoassay (ELISA) kits were obtained from R&D. MitoSOX, MitoTracker Green, MitoTracker Deep Red, and mouse recombinant TNF-α were purchased from Invitrogen. Annexin V-FITC apoptosis detection kit was obtained from BD Bioscience. Anti-human caspase-1 (p10), anti-ASC, anti-phospho-ERK and anti-β-actin antibodies were purchased from Santa Cruz. Anti-mouse IL-1β antibody was obtained from R&D. Anti-mouse caspase-1 (p20) and anti-NLRP3 antibodies were from Adipogen. Anti-Drp1 and anti-JNK1/2 antibody were purchased from BD Biosciences. Anti-phospho-JNK1/2 antibody was obtained from Invitrogen. Anti-VDAC1 antibody was obtained from Abcam. All other antibodies detecting human IL-1β, caspase-3, ERK, and IκB were obtained from Cell Signaling.

### Cell culture

Mouse bone marrow-derived macrophages (BMDMs) were prepared from C57BL/6, *Nlrp3*^−/−^ or *Atg5*^−/−^ mice as described previously^41^. All mice were maintained under specific pathogen-free conditions. Protocols for the animal experiments were approved by the Institutional Ethical Committee, Yonsei University College of Medicine. All the experiments involving BMDM preparation were performed in accordance with the approved guidelines of the Institutional Ethical Committee. Immortalized NLRP3-reconstituted BMDMs (N1–8) were kindly provided by Dr. E. S. Alnemri (Thomas Jefferson University). To knockdown Drp1 expression, we used lentiviral particles containing non-targeting (scrambled) or targeting Drp1 shRNA to infect mouse BMDMs. Cells stably expressing shRNA were then cloned by puromycin selection. All BMDMs were maintained in L929-conditioned DMEM supplemented with 10% FBS and 100 U/ml penicillin/streptomycin. THP-1 cells were cultured in RPMI1640 supplemented with 10% FBS, 2 mM glutamine, 10 mM HEPES, 1 mM sodium pyruvate, 0.05 mM 2-ME and 100 U/ml penicillin/streptomycin. To knockdown Drp1 or Opa1 expression, lentiviral particles containing non-targeting, Drp1-targeting or Opa1-targeting shRNA were used to infect THP-1 cells.

### Determination of cell death

To induce apoptotic, necroptotic, or pyroptotic cell death, cells were treated with TNF-α (30 ng/ml) and cycloheximide (CHX, 0.4 μg/ml) with or without zVAD-fmk (20 μM) for 20 h, or cells were primed with LPS (0.25 μg/ml, 2 h), followed by treatment with nigericin (5 μM, 45 min). Cell death was then determined by the extracellular release of lactate dehydrogenase (LDH) using a CytoTox96 non-radioactive cytotoxicity assay kit (Promega). LDH release was calculated as [extracellular LDH/(intracellular LDH + extracellular LDH) × 100].

### Immunoblot analysis

Immunoblotting, co-immunoprecipitation and subcellular fractionation experiments were performed as described previously^8^. All the immunoblots are representative images of at least three independent experiments.

### Observation of mitochondrial morphology

For confocal microscopy, cells were stained with MitoTracker Red (Invitrogen) and Hoechst 33258 (Sigma) according to the manufacturer’s protocol, and examined using a confocal microscope (LSM700, Carl Zeiss). In some experiments, immunofluorescence assays were performed using anti-Tom 20 antibody to observe mitochondria as described previously^20^. For transmission electron microscopy (TEM), cells were fixed with 2% glutaraldehyde-paraformaldehyde in 0.1 M phosphate buffer, pH 7.4, for 2 h. After washing, cells were post-fixed with 1% OsO_4_ in 0.1 M phosphate buffer for 2 h and dehydrated in ascending gradual series (50~100%) of ethanol. Specimens were embedded using a Poly/Bed 812 kit (Polysciences, Inc.). Seventy-nanometer-thin sections were stained with uranyl acetate and lead citrate. Stained sections were then observed using a JEM-1011 (JEOL) transmission electron microscope.

### Assay of NLRP3 inflammasome activation

Mouse BMDMs were primed with LPS (0.25 μg/ml) for 3 h, followed with ATP (1–2.5 mM, 30–40 min) or nigericin (2.5 μM, 40 min) treatment. THP-1 cells were differentiated with PMA (0.4 mM) for 3 h, and the next day cells were stimulated with LPS (0.5 μg/ml) or Alum (250 μg/ml) for 6 h. The culture supernatants were precipitated as described previously^42^, and were then immunoblotted with anti-caspase-1 or IL-1β antibody, or assayed for extracellular IL-1β using ELISA. To check the oligomerization of ASC, chemical cross-linking was performed using disuccinimidyl suberate (DSS), according to previously described methods^43^.

### Quantification of mRNA production

To measure mRNA production, total cellular RNA was isolated using the TRIzol reagent (Invitrogen) and reverse transcribed using PrimeScript™ RT Master Mix (Takara) according to the manufacturer’s instructions. Template DNA was amplified by quantitative real-time PCR using SYBR *Premix Ex Taq*™II (Takara). Primers were as follows: 5′-ATG CTG CTT CGA CAT CTC CT-3′ and 5′-AAC CAA TGC GAG ATC CTG AC-3′. (*Nlrp3*); 5′-AAC TTT GGC ATT GTG GAA GG-3′ and 5′-ACA CAT TGG GGG TAG GAA CA-3 (*GAPDH*).

### *Salmonella* infection

*S. typhimurium* (kindly gifted from Dr. Yoon SS, Yonsei University College of Medicine) were grown overnight at 37 °C with aeration, and then diluted (1:20) and grown for additional 2 h. BMDMs were infected with *S. typhimurium* at the indicated MOI (multiplicity of infection) for 30 min, washed three times to remove extracellular bacteria, and incubated with the gentamicin (100 μg/ml)-containing medium for 150 min before harvest.

### Determination of mitochondria-derived products and mitochondrial damage

To determine mROS production, cells were stained with MitoSOX (Invitrogen) according to the manufacturer’s protocol. The fluorescence of the cells was then monitored by flow cytometer (FACSVerse, BD). To measure cytosolic mtDNA, cells were lysed using a 21G syringe in NP-40-free buffer A, and cytosolic fractions were prepared as described in Subcellular fractionation subsection. Then, DNA was isolated from the normalized cytosolic fraction and the copy number of the gene encoding cytochrome *c* oxidase I was determined by quantitative real-time PCR using the following primers as described previously^7^. 5′-GCCCCAGATATAGCATTCCC-3′ (forward) and 5′-GTTCATCCTGTTCCTGCTCC-3′ (reverse). To determine mitochondrial damage, cells were costained with MitoTracker Deep Red and MitoTracker Green and then analyzed by flow cytometry. All the flow cytometry data are representative of at least three independent experiments.

### Statistical analysis

All values were expressed as the mean ± SEM of individual samples or independent experiments. Data were analyzed using the Student’s *t* test, and *p* values ≤ 0.05 were considered significant.

## Additional Information

**How to cite this article**: Park, S. *et al.* Defective mitochondrial fission augments NLRP3 inflammasome activation. *Sci. Rep.* **5**, 15489; doi: 10.1038/srep15489 (2015).

## Supplementary Material

Supplementary Information
