# Supplementary material for: Defective mitochondrial fission augments NLRP3 inflammasome activation
Source: Sci Rep. 2015 Oct 22;5:15489. doi: 10.1038/srep15489 (PMC4614538; doi:10.1038/srep15489)
Supplement: Supplementary Information [file srep15489-s1.pdf]

Supplementary Figures

Defective mitochondrial fission augments NLRP3 inflammasome activation

Sangjun Park[1], Ji-Hee Won[1], Inhwa Hwang[1], Sujeong Hong[1], Heung Kyu Lee[2] and Je-Wook Yu[1]

[1]Department of Microbiology, Institute for Immunology and Immunological Diseases, Brain Korea 21 PLUS Project for Medical Science, Yonsei University College of Medicine, Seoul 120-752, Korea,

[2]Laboratory of Host Defenses, Graduate School of Medical Science and Engineering, Korea Advanced Institute of Science and Technology, Daejeon 305-701, Korea

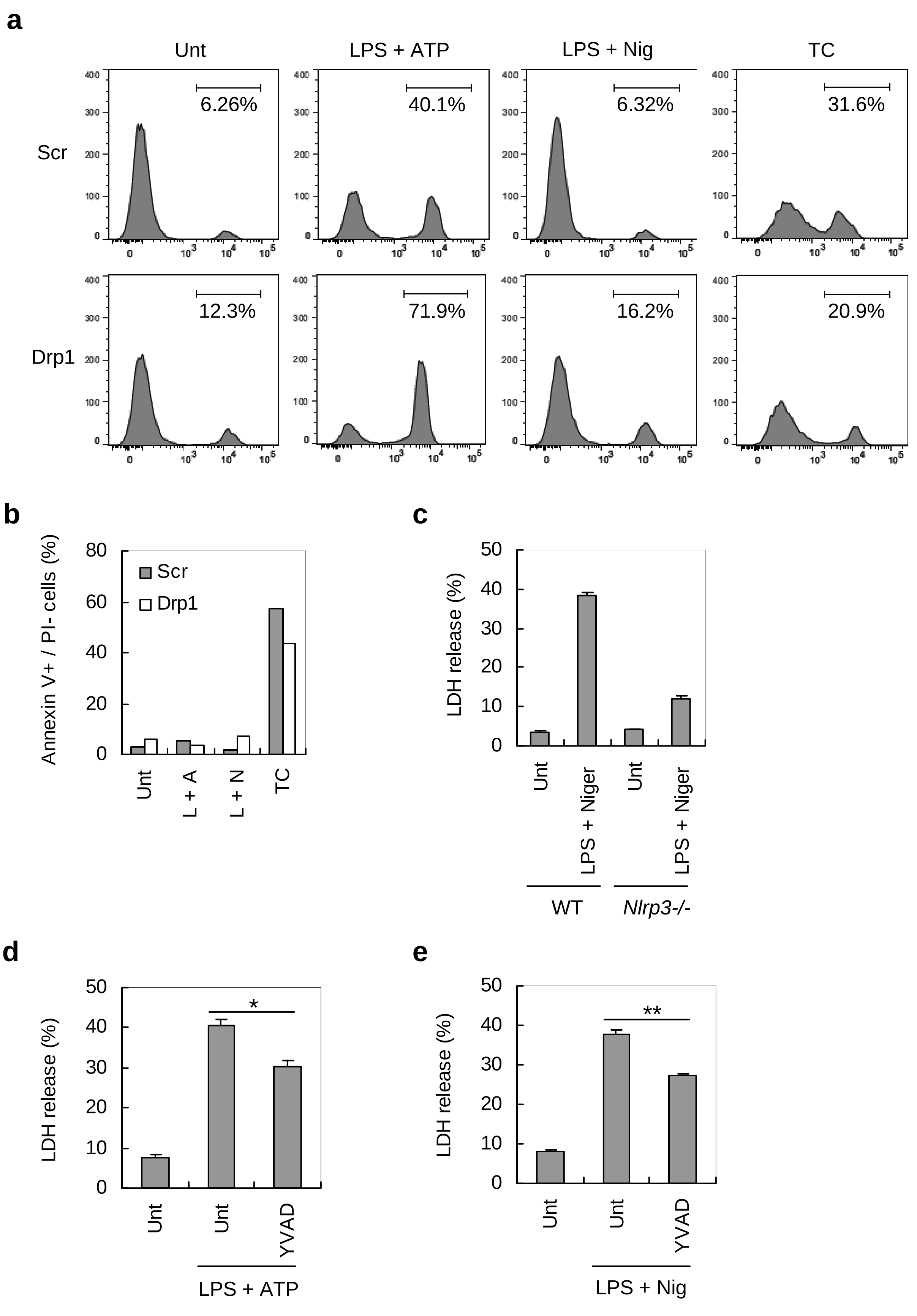
a
Unt
LPS + ATP
LPS + Nig
TC
Scr
6.26%
40.1%
6.32%
31.6%
Drp1
12.3%
71.9%
16.2%
20.9%
b
Annexin V+ / PI- cells (%)
Scr
Drp1
Unt
L + A
L + N
TC
c
LDH release (%)
Unt
LPS + Niger
Unt
LPS + Niger
WT
Nlrp3-/-
d
LDH release (%)
*
Unt
Unt
YVAD
LPS + ATP
e
LDH release (%)
**
Unt
Unt
YVAD
LPS + Nig

Supplementary Figure S1. Knockdown of Drp1 leads to augmented NLRP3-activating stimulations-mediated cell death. (a, b) ShScr or shDrp1 BMDMs were treated with LPS, followed by ATP (L + A) or nigericin (L + N) treatment, or treated with TNF-α and CHX (TC). Cells were stained with propidium iodide (PI) (a) or Annexin V-FITC/PI (b), and then analyzed by flow cytometer. Relative PI-positive cells (a) or Annexin V-positive/PI-negative cells (b) were plotted. (c) Wild-type or *Nlrp3*-deficient BMDMs were treated with LPS, followed by nigercin treatment. Cell death was then determined by LDH release assay. (d, e) ShDrp1 BMDMs were treated with LPS in the presence of YVAD-cmk (20 μM), followed by ATP (d) or nigericin (e) treatment as indicated. Cell death was determined by LDH assay. Asterisks indicate significant differences (n = 7, * p<0.0005; n = 7, ** p<0.0001).

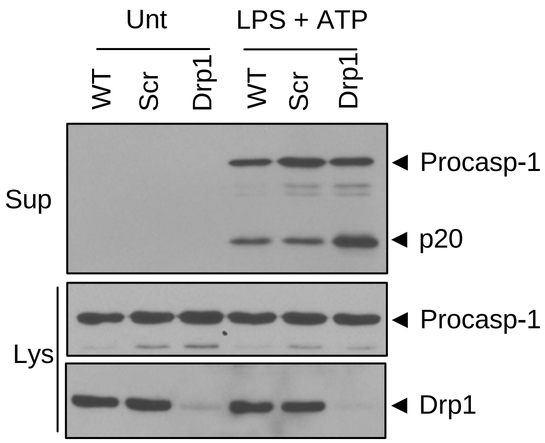

Supplementary Figure S2. Knockdown of Drp1 leads to augmented NLRP3-dependent caspase-1 activation. Wild-type, shScr or shDrp1 BMDMs were untreated or treated with LPS, followed by ATP treatment (2 mM, 45 min). Culture supernatants (Sup) or soluble lysates (Lys) were immunoblotted as indicated antibodies.

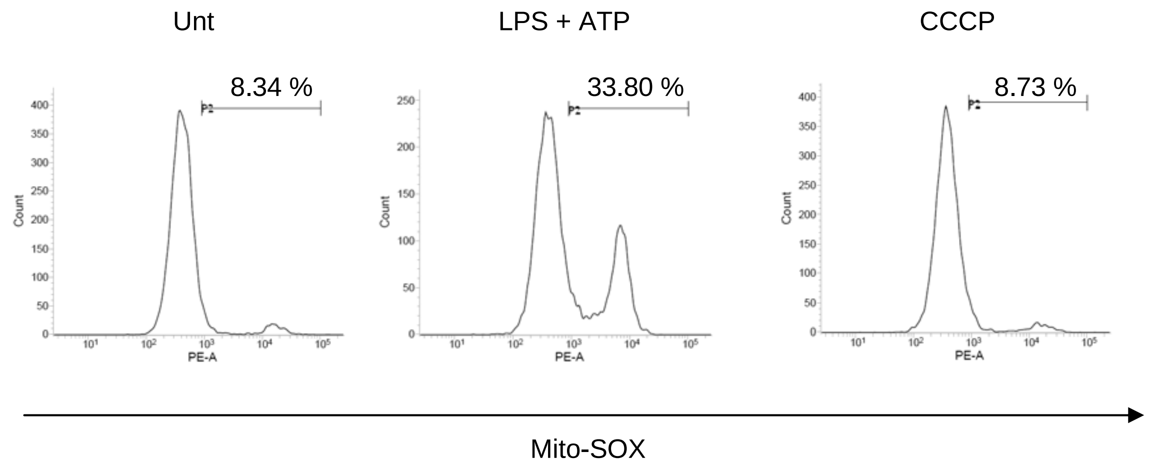

Supplementary Figure S3. CCCP did not promote the production of mitochondrial ROS. Wild-type BMDMs were treated with LPS, followed by ATP treatment, or treated with CCCP (10 μM, 2 h). Cells were stained with MitoSOX and analyzed by flow cytometer.

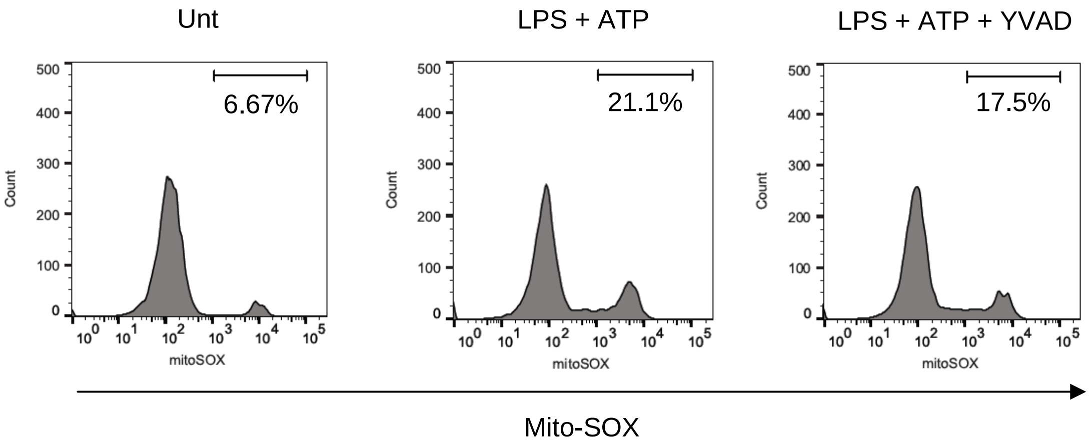

Supplementary Figure S4. Inhibition of caspase-1 partially attenuates LPS/ATP-triggered mitochondrial ROS production. Wild-type BMDMs were treated with LPS, followed by ATP treatment in the presence of YVAD-cmk (20 μM) as indicated. Cells were stained with MitoSOX and analyzed by flow cytometer. Representative histograms among four-independent experiments were displayed.

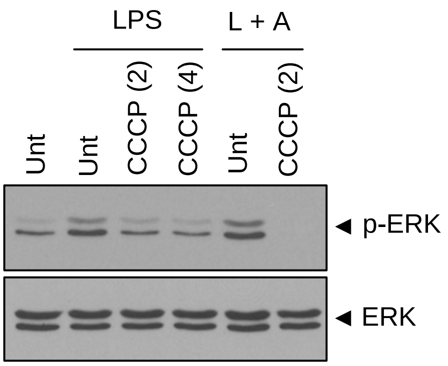

Supplementary Figure S5. CCCP attenuates LPS-mediated ERK phosphorylation. Wild-type BMDMs were untreated or treated with LPS in the presence or absence of CCCP pretreatment (5 μM) for 2 h or 4 h, followed with ATP as indicated.

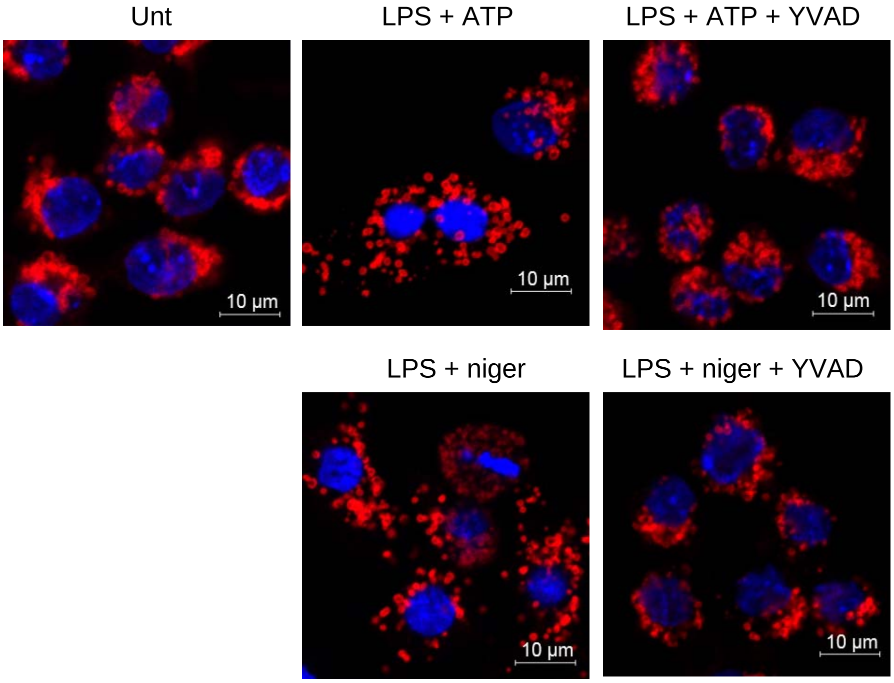

Supplementary Figure S6. Caspase-1 inhibitor blocks mitochondrial fragmentation by NLRP3 inflammasome stimulators. Wild-type BMDMs were treated with LPS in the presence or absence of YVAD (20 μM), followed by ATP or nigericin treatment as indicated. Cells were then stained with anti-Tom20 antibody, and observed by confocal microscope. Red and blue signal represent mitochondrial and nuclear fluorescence, respectively. Scale bars, 10 μm.
